# Navigating ambivalence: perspectives on healthy eating habits and readiness for change among Swedish men with prediabetes

**DOI:** 10.3389/fcdhc.2026.1793053

**Published:** 2026-05-12

**Authors:** Åsa Carlsund, Åsa Hörnsten, Fatima Hoosen, Stephen Amoah, Julia Otten

**Affiliations:** 1 Umeå University, Umeå, Sweden; 2 University of Cape Town, Cape Town, South Africa; 3 University of Bonn, Bonn, Germany

**Keywords:** experiences, individualized diet advice, men, prediabetes, qualitative research, Sweden

## Abstract

**Background:**

Type 2 diabetes accounts for over 90% of all diabetes cases and continues to rise globally, with projections reaching 853 million adults by 2050. Prediabetes, a key risk state, offers a critical window for prevention, as lifestyle changes can reduce progression to type 2 diabetes by nearly 50%. Despite clear guidelines emphasizing diet and physical activity, adherence remains challenging due to socioeconomic, cultural, and behavioral barriers.

**Aim:**

The aim of this study was to explore men’s attitudes toward managing their diet and lifestyle after being diagnosed with prediabetes.

**Methods:**

A qualitative study used individual and group interviews with 21 Swedish men aged 60–72 with prediabetes, recruited from the Swedish CArdioPulmonary bioImage Study (SCAPIS) cohort via convenience sampling. Data collected from May to August 2025 through Microsoft Teams included open-ended questions. Interviews were audio-recorded and transcribed. Analysis followed Graneheim and Lundman’s qualitative content analysis.

**Results:**

The men recognized the importance of healthy habits but doubted they could maintain lasting change. This ambivalence was caused by personal limitations and a focus on short-term goals. Some men sought support from family or friends, moving from contemplation to preparation. Learning from successes and setbacks, they gained a more realistic view of their ability to change. The analysis identified one main theme, “Grasping the message about healthy habits but still doubting one’s ability to change”, and two additional themes, each with three subthemes.

**Conclusion:**

Men with prediabetes know how to eat healthy but struggle to turn knowledge into habits. Emotional distress, ambivalence, and triggers often undermine motivation. Short-term goals like weight loss dominate over long-term strategies. Yet, men seek social support and engaging activities, showing readiness for change.

**Implications:**

Interventions should target psychological barriers, use social networks, and offer practical strategies for lasting lifestyle changes.

## Background

Type 2 diabetes (T2D) is a rapidly growing global public health challenge, accounting for over 90% of all diabetes cases worldwide (1). In 2024, an estimated 589 million adults aged 20–79 were living with diabetes, and projections suggest this number may increase to 853 million by 2050 (2). In addition, approximately 1.1 billion adults worldwide have impaired glucose tolerance (IGT) or impaired fasting glycemia, placing them at a substantial risk of developing T2D (1). The pathogenesis of T2D is multifactorial and involves a complex interplay between genetic predisposition, insulin resistance, and beta-cell dysfunction. Importantly, modifiable lifestyle factors, particularly diet, physical inactivity, and obesity, play a central role in disease onset and progression (3, 4). Prediabetes, defined by elevated blood glucose levels that do not yet meet diagnostic criteria for diabetes, represents a critical opportunity for preventive intervention. Without targeted lifestyle modification, a substantial proportion of individuals with prediabetes will progress to T2D within a few years (5, 6). Modifiable risk factors for prediabetes also exhibit distinct gender-related patterns. In men, abdominal obesity, dyslipidemia, smoking, and alcohol consumption are more strongly associated with prediabetes risk, whereas in women, hypertension and poor dietary quality appear to be more prominent predictors (7).

Among the different types of prediabetes, IGT is of particular clinical relevance due to its strong association with peripheral insulin resistance and ongoing beta-cell dysfunction (8–10). IGT is characterized by elevated 2-h plasma glucose levels after an oral glucose tolerance test (OGTT), and individuals with IGT face a markedly elevated risk of developing T2D and cardiovascular disease (8, 9). Compared with isolated impaired fasting glucose (IFG), IGT is more closely linked to lifestyle factors like physical inactivity and poor dietary habits, highlighting its importance as a primary target for preventive interventions (10). Previous studies have confirmed that lifestyle modifications, including dietary changes and increased physical activity, can significantly reduce the incidence of T2D in high-risk populations (11, 12). For example, a meta-analysis found that lifestyle interventions reduced the risk of developing T2D by 47% (RR = 0.53, 95% CI: 0.41–0.67) (13). Effective dietary strategies for both men and women include higher intake of fibers, whole grains, vegetables, and healthy fats, together with reduced consumption of refined sugars and saturated fats (3, 14, 15).

Health behavior change has commonly been conceptualized as a dynamic and nonlinear process influenced by motivational, cognitive, and contextual factors. The Transtheoretical Model (TTM) describes behavior change as progressing through stages of readiness, ranging from contemplation to action and maintenance, highlighting ambivalence and relapse as natural components of change (16). In parallel, Self-Determination Theory (SDT) emphasizes the role of autonomous motivation, perceived competence, and relatedness in sustaining health behaviors over time (17, 18). From a Social Cognitive Theory (SCT) perspective, behavior change is shaped through reciprocal interactions between personal factors, behavior, and the social environment, with self-efficacy playing a central role (19). However, although the effectiveness of lifestyle interventions is well established, adopting and maintaining these changes is often difficult, highlighting the importance of understanding the behavioral processes behind health-related change.

Together, these frameworks provide a useful lens for understanding how individuals may recognize the need for lifestyle change yet struggle to translate awareness into sustained action, particularly in the presence of emotional, social, and environmental barriers. Although the current study was not specifically designed to test behavioral theories, the TTM, SDT, and SCT served as guiding frameworks to interpret the findings. These theories helped explain men’s expressed ambivalence, perceived self-efficacy, and shifting motivation related to dietary and lifestyle changes.

To inform clinical practice, several organizations have provided updated dietary guidelines for individuals at risk of T2D. The International Diabetes Federation (IDF) and the American Diabetes Association (ADA) advocate a person-centered approach that includes evidence-based eating patterns, weight management, and metabolic goals (3, 20). Despite these recommendations, challenges remain in implementation. Nevertheless, adherence to dietary advice is often hindered by socioeconomic factors, cultural preferences, and limited access to healthy foods. Accordingly, person-centered nutrition strategies and behavioral support are increasingly recognized as a key component of effective diabetes prevention programs. Socioeconomic barriers may include limited financial resources, time constraints due to work, and competing everyday demands, which limit the ability to prioritize dietary change (3). Cultural barriers can involve entrenched food traditions, social norms around eating and alcohol consumption, and gendered expectations related to masculinity and food practices (21). Behavioral barriers include ambivalence toward change, low perceived self-efficacy, and difficulties maintaining motivation over time, particularly when lifestyle changes conflict with established routines or social contexts (3). Additionally, psychological barriers are important and may include hesitance toward change, emotional reactions to risk information, low perceived self-efficacy, and challenges in integrating lifestyle advice into one’s self-image and daily habits. These barriers can lower motivation and hinder long-term behavior change, even when awareness and knowledge are there (21, 22).

In summary, the global burden of T2D continues to rise, driven largely by preventable lifestyle factors. Early identification of individuals at elevated risk, combined with targeted dietary interventions and supportive care, represents a promising strategy to reduce disease incidence and improve long-term health outcomes. Despite increasing quantitative evidence on lifestyle interventions for prediabetes, there is limited qualitative research exploring how men interpret, emotionally process, and negotiate dietary and lifestyle advice after a diagnosis of IGT. Existing studies rarely focus solely on men or examine the early post-diagnosis phase, during which ambivalence, motivation, and readiness for change are developed. A deeper understanding of men’s lived experiences is therefore necessary to inform person-centered and gender-responsive prevention strategies. Insight is needed into how socioeconomic conditions, cultural norms, and behavioral challenges are experienced and negotiated in everyday life following a prediabetes diagnosis. In this context. Therefore, the aim of this study was to explore men’s attitudes toward managing their diet and lifestyle after being diagnosed with prediabetes.

## Materials and methods

### Design

This study employed a qualitative design, reporting findings from group interviews complemented by individual interviews with men diagnosed with prediabetes. The data were analyzed using qualitative content analysis (23, 24). The study forms a part of a larger interdisciplinary project, and to ensure gender-specific insights, data collection, analysis, and reporting were conducted in parallel but separately for men and women.

### Participants

Men participated in the Swedish CArdioPulmonary bioImage Study (SCAPIS) in Umeå, Sweden between April 2024 and April 2025, and had undergone an OGTT. Men with glucose levels between 8.9 and 12.1 mmol/L were diagnosed with prediabetes (25), received written information about it, and were advised to seek follow-up blood glucose testing after 1 year. Men diagnosed with prediabetes within SCAPIS who had provided consent to be contacted for future research were invited to participate in the present study. The inclusion criteria for this study were therefore explicitly defined as: (1) men participating in SCAPIS from April 2024 to April 2025; (2) having completed an OGTT during the designated study period; (3) glucose levels between 8.9 and 12.1 mmol/L indicating prediabetes; and (4) having consented to be recontacted for future research. The first 50 men on the SCAPIS recontact list who met all inclusion criteria constituted the entire eligible pool at the study site at the time of recruitment.

Calls were repeated twice if unanswered. Of the 26 men who responded, 16 agreed to participate in a group interview, and four group interviews were conducted, each with 4 men. Five men were unable to attend the scheduled group interviews for various reasons and were therefore offered individual interviews. A purposive sampling strategy was applied. The final sample of 21 (**Table 1**) participants aligns with methodological guidelines, which state that three to five groups are sufficient to achieve information richness and depth (26, 27). Twelve men lived in rural areas and nine resided in small cities. Fourteen participants were retired, and seven were currently employed. Participant ages ranged from 60 to 72 years (mean = 67). The narrow age range mirrors the underlying SCAPIS population from which participants were recruited, as the eligible women who met all inclusion criteria were predominantly in this age group. Accordingly, the age profile of the final sample reflects the characteristics of the available population.

**Table 1:** Descriptions of the participants (n = 21).

Type of interview Group Individual Age range (mean)	16 5 60–72 (67)
Work status (*n*)	
Retired	14
Employed	7
Place of living (*n*)	
Small city	9
Rural	12

### Data collection

Participants were recruited using convenience sampling. Prior to participation, all men received both verbal and written information about the study’s aims and procedures and provided informed consent. Data collection was conducted between May and August 2025. Four group interviews, each comprising four men, were conducted and lasted between 50 and 60 min. For men (*n* = 5) who were unable to attend the scheduled group interviews, individual interviews were offered, with durations ranging from 22 to 36 min.

All interviews were conducted and recorded via Microsoft Teams. To capture areas relevant to the study aim, including participants’ experiences of dietary habits, lifestyle changes, perceived challenges, and support needs following a prediabetes diagnosis, a semi-structured interview guide with contextual background questions and open-ended questions was used (**Appendix 1**). Example questions included the following: *“Have you previously tried to change your diet?”*, *“What type of dietary changes did you make?”*, *“What are your thoughts on the relationship between diet and the risk of developing type 2 diabetes?”*, and *“How would you experience receiving tailored dietary advice?”* Following data collection, the recordings were reviewed and transcribed verbatim. Non-verbal expressions, such as laughter, pauses, or expressions of agreement or hesitation, were noted and included in the analytic material.

The interviews were conducted by members of the research team with professional backgrounds in nursing (ÅC and ÅH) and experience in qualitative research. The interviewers were not involved in the men’s clinical care and had no prior personal or therapeutic relationship with the men. The men were recruited through the SCAPIS cohort, and the researchers had no direct involvement in recruitment decisions within the cohort.

### Analysis

The transcribed texts were analyzed using qualitative content analysis (23, 24). During the de-contextualization phase, the texts were read thoroughly and discussed among researchers to gain a general understanding before starting to identify and divide the text into meaning units, answering the aim. These meaning units were subsequently condensed while preserving their core meaning. The condensed units were then assigned codes, facilitating comparison and organization. During the re-contextualisation phase, the codes were compared and grouped based on their similarities and differences and organized into preliminary initial themes. Through further interpretation and abstraction, these preliminary themes were organized into higher-order themes, and an overarching main theme gradually emerged. Although described sequentially, the analytic process was iterative and dynamic, involving continuous movement between the raw data, codes, and different levels of abstraction. Throughout the analytic process, attention was paid not only to the presence of codes, but also to how the men emphasized, elaborated on, and emotionally positioned different experiences. Interpretative weight was assigned based on the depth, coherence, and recurrence of meaning across interviews rather than frequency counts. Patterns that appeared across both group and individual interviews were treated as analytically significant.

Theme labels were refined throughout the analytic process, which continued into the writing phase of the Results section. Thematic saturation was monitored continuously. As analysis progressed, no new codes or themes appeared in the final interviews. This consistency across data sources (group and individual interviews) indicated enough informational power to meet the study aim.

### Trustworthiness

Trustworthiness was addressed by evaluating credibility, dependability, and transferability. Qualitative content analysis was selected as it is a systematic and well-established method, suitable for capturing variations in experiences and meanings (23, 24), and one with which the research team has prior experience. To preserve the integrity of the men’s accounts, both decontextualization and recontextualization were applied throughout the analysis.

Credibility was strengthened by the inclusion of representative quotations illustrating men’s experiences, as well as disconfirming cases to reflect variation within the data. Interpretations were discussed among the co-researchers until consensus was reached, ensuring that the findings reflected the most plausible meanings of men’s perspectives. By providing detailed descriptions of the participants and study context, the study enables readers to assess the transferability of the findings to similar populations and settings. The inclusion of quotations allows readers to assess the relevance of the findings to other settings.

Reflexivity was maintained throughout the research process. The researchers’ backgrounds in nursing and qualitative research helped them stay sensitive to the men’s narratives, while regular analytic discussions challenged preconceptions and enhanced interpretative rigor.

### Ethical considerations

The study followed the ethical standards outlined in the Helsinki Declaration and the World Medical Association. Ethical approval was obtained from the Swedish Ethical Review Authority (Dno: 2025-00193-01-69890). Participation was voluntary, with all individuals providing written informed consent. Measures were taken to protect their integrity during the research. Each consent was connected to a unique digit assigned to the men, which was retained throughout all research phases.

## Results

The analysis that resulted in one main theme and two themes, each with three sub-themes, is presented in **Table 2** and is further detailed in the text, along with exemplifying original quotes. Participants’ accounts were closely connected to their personal life circumstances, such as family illnesses and their own health conditions. These contextual factors affected how dietary advice and lifestyle changes were understood, prioritized, and emotionally managed.

**Table 2:** Main theme, themes, and subthemes.

Main theme	Themes	Subthemes
Grasping the message about healthy habits but still doubting one’s ability to change	Awareness of unhealthy habits and a preparation for action	Getting new insights into habits and personal risk Accepting the need for improved health habits Acquiring help to prepare for change
	Recognition of personal limitations and a reluctance toward long-term change	Learning from achievements and setbacks Accepting one’s weak points Focusing on short-term goals only

### Grasping the message about healthy habits but still doubting one’s ability to change

The main theme indicates that men were aware of the healthcare provider’s message about healthy habits. At the same time, they revealed doubts about their ability to initiate and sustain behavioral changes over time, often expressed through reflections on previous failed attempts or fluctuating motivation. Men actively sought support from others, like family or friends, to prepare for change, suggesting a transition from contemplation to readiness for action. Men described learning from both successes and setbacks, gradually acknowledging their vulnerabilities and developing a more realistic and self-reflective understanding of their capacity for change.

### Awareness of unhealthy habits and a preparation for action

The first theme comprises three subthemes: “Getting new insights into habits and personal risk”, “Accepting the need for improved health habits”, and “Acquiring help to prepare for change”. Each subtheme is described below.

#### Getting new insights into habits and personal risk

Men’s reflections revealed that awareness of a hereditary predisposition to T2D was often present but emotionally distant until activated by concrete experiences. As one participant explained: “My mom and dad both developed type 2 diabetes. So you know that’s probably the case, and you have to take those things with you, because it’s better to be safe than sorry” (GI1).

This latent awareness often remained in the background unless triggered by specific events. Personal or family experiences of diabetes-related complications frequently acted as catalysts for deeper reflection. For example, witnessing a relative experience a severe complication, such as an amputation, served as an emotional turning point, increasing concern and prompting a reassessment of personal risk. These experiences appeared to shift risk perception from a theoretical understanding to a more personal and immediate concern, occasionally motivating a desire to engage in preventive actions.

#### Accepting the need for improved health habits

Men described a clear awareness of their current health status and an intention to improve their health habits. Health concerns such as high cholesterol and the risk of developing T2D often motivated deliberate self-management efforts. These efforts included adopting healthier eating practices, such as following the plate model, reducing fat intake, and selecting specific foods—seed crispbread, unsweetened graham rusks, lentils, and sprouts—that were perceived as both health-promoting and satiating. Some men also introduced stress-management strategies, for example, relaxation routines, after linking stress to elevated blood pressure. Regular meal schedules and evening routines were described as structured attempts to maintain consistency over time.

At the same time, these deliberate strategies were repeatedly challenged by habitual behaviors that undermined the men’s intentions. Evening snacking, particularly the habitual consumption of crisps, was described as difficult to resist, even among those who were otherwise committed to dietary change. This contrast between intention and action was reflected in narratives that combined determination with fear of future illness and a sense of ongoing struggle. As one participant explained: “Yes, I will, I can avoid it. I will avoid it. And yes, that was simply my motivation. What a miserable life those with type 2 have all the time, they have to be looked after and checked on, and they have to have food in front of them all the time. And then I thought, I don’t want to feel that pressure all the time” (IW3).

Despite strong motivation, sustaining behavioral change required continuous effort. Men emphasized persistence and self-discipline as necessary but fragile resources, easily disrupted by routines, stress, or fatigue. Thus, acceptance of the need for improved health habits was characterized not by stable change, but by an ongoing negotiation between purposeful self-management and recurring self-sabotaging practices.

#### Acquiring help to prepare for change

Men’s accounts showed that preparing for dietary change involved identifying concrete everyday situations that either supported or undermined their intentions. One commonly described strategy was avoiding grocery shopping when hungry, as this situation was associated with impulsive, less healthy food choices. Such adaptations were described as practical ways of reducing self-sabotage and creating conditions that made healthier choices easier to sustain.

To support these efforts, men used practical tools such as recipe folders to facilitate meal planning and increase dietary variation. These tools were described as helping men translate general advice into everyday practice. Shared responsibility for meal preparation was also emphasized, often involving reliance on a partner’s nutritional knowledge and cooking skills. This collaborative approach was described as providing both practical structure and emotional encouragement, enabling households to maintain healthier routines over time.

“Maybe a man needs a kick in the butt for one reason or another … we have to try to encourage each other, help each other to stay focused” (IW2).

Dietary changes were consistently framed in terms of what was considered most practical and manageable in everyday life. Men described gradually replacing unhealthy alternatives with healthier ones rather than making abrupt changes. However, some foods, particularly vegetables such as carrots and cabbage, were described less as enjoyable meals and more as something to be consumed for health reasons, almost as a form of treatment rather than food:

“You eat it because you know it’s good for you, not because you really want to” (GI1).

Convenience played a central role in sustaining new habits. Pre-cut vegetables stored in the fridge were described as making healthy choices easier to maintain, particularly on busy days. Foods that were initially unfamiliar or perceived as unappealing, such as lentils and vegetarian dishes, became more accepted over time as men discovered that they could be both filling and satisfying. Despite these changes, traditional staples, such as meat, potatoes, and bread, often remained central to meals.

Across these accounts, men emphasized the importance of seeing tangible results from their efforts, such as weight loss or improved wellbeing, to maintain motivation and commitment. Progress appeared to confirm that the effort was worthwhile and encouraged continued adherence to healthier choices. In this context, tailored and individualized advice was described as particularly important, as it helped men experience lifestyle changes as personally meaningful rather than generic:

“I think individualized advice would be good … if it’s tailored to the individual, it becomes more meaningful, and it becomes easier to follow” (IW1.)

### Recognition of personal limitations and a reluctance toward long-term change

The second theme also comprises three subthemes: “Learning from achievements and setbacks”, “Accepting one’s weak points”, and “Focusing on short-term goals only”.

#### Learning from achievements and setbacks

The men described a complex relationship between self-image, long-term habits, and maintenance of healthy routines. They experienced shifting success and relapse influenced by emotional states and stress related to work. A key struggle was how they saw themselves and their current physical condition. One man mentioned that discomfort in daily tasks, such as tying shoes, caused emotional distress. However, sustaining health changes was described as difficult. For example, a man who lost 15 kg and then regained 10 kg felt discouraged and doubted his own capability. Work changes also affected health behaviors; for example, shifting from active to sedentary jobs reduced energy and made healthy routines more difficult. Furthermore, having more control over routines, especially during retirement or less busy periods, helped men adhere to their health goals. Seasonal changes, especially winter, made adherence more challenging, with snow and cold weather cited as major obstacles for outdoor activities and exercise. While traditional gyms were generally viewed as unappealing, interests in accessible activities, such as walking football and senior table tennis, was expressed. Overall, attempts to adopt healthier behaviors were fragile and vulnerable to disruptions. The men tried to follow a balanced diet and exercise regularly, but faced obstacles such as a lack of motivation, mental challenges, and economic barriers. A man said he tried to exercise a few times weekly, but things didn’t work out as planned.

“I’m the kind of person who, eventually becomes desperate enough when I’m not really happy with myself, like when it was too difficult to tie my shoes and stuff like that. Last year, I lost 15 kg. But then, since then, I’ve gained 10 kg again, and now it feels like no … But that’s how we can feel, I guess, but I’m a little disappointed in myself in the sense that I don’t feel like I really have that stamina in any way, or that I can’t do it seriously, but instead it’s a bit up and down. I feel like it’s a bit pointless” (GI2).

#### Accepting one’s weak points

The men felt a deep disappointment in themselves, especially about their perceived inability to maintain consistent longer-term healthy habits. They mentioned a lack of endurance and self-discipline, which led to feelings of low self-efficacy. This was often paired with a sense of uselessness and a tendency to give up. While they acknowledged their ability to lose weight, they also described a recurring cycle of losing and regaining weight. These ups and downs led them to believe their efforts were ultimately pointless, which in turn reduced their motivation. They also discussed how their work lives and daily routines impacted their capacity to maintain healthy habits. During busier periods, managing mealtimes and maintaining physical activity became more difficult. Conversely, calmer or more structured times made it easier to keep track of their lifestyles and follow healthier routines. Men described how life transitions, especially retirement, changed their daily routines in ways that could either promote or hinder lifestyle changes. For some, having more control over their time after retirement made it easier to focus on physical activity and regular meals. For others, the loss of structure that work routines used to provide led to less motivation and more sedentary habits.

A common theme was feelings of guilt and poor self-esteem. They described themselves as lazy or physically weak, often feeling guilty for not exercising enough or for lacking a gym membership. Some included walking to work to stay active, but they often felt it was not enough. Physical limitations and a perceived decline in fitness were seen as obstacles to remaining active.

“Yes, I mean, I have trouble bending down and tying my own shoes, it’s unbelievable!!! How am I going to cope with getting from this to being able to move freely and without limitations?” (IW5).

Men, though, showed a strong understanding of their personal vulnerabilities and triggers related to eating and drinking habits. However, these triggers differed among individuals. Eating in certain situations, especially at home, was often seen as a challenge. Snacking frequently occurred with routines like coffee breaks or alongside meals. However, some men felt confident managing their overall diet, such as enjoying a sweet treat with their morning coffee. Alcohol intake was described as moderate and dependent on the situation. They made a distinction between everyday habits and special occasions, noting that alcohol often followed routines, like having some glasses of wine to relax on weekends.

“I should drink more water and less of everything else, so to speak. But on weekends, we often have a few glasses of wine. However, it becomes a habit to pour wine as soon as you want to relax, but we try to think a little bit and stick to one glass” (GI1).

#### Focusing on short-term goals only

The men described a pattern of focusing on short-term goals rather than adopting a long-term perspective on health behavior changes. This approach was closely connected to physical discomfort and dissatisfaction with their bodies, with the primary goal being to lose weight rather than to build sustainable habits. They acknowledged that seeking quick progress rather than committing to lifelong change may be the wrong approach. They also reflected on how early life experiences influenced their current attitudes toward food and body image. Childhood memories, such as teasing from peers, were seen as forming lasting negative impressions. These memories often led to times of urgency and pressure to alter one’s body, such as through various diets or other health advice from friends, family, or commercials.

“Perhaps it’s the foundation that’s a little crazy from the start, perhaps I am chasing quick profits instead of thinking that it’s a lifelong commitment” (IW4).

Physical activity was generally viewed as necessary but was often discussed vaguely and lacked structured planning. While men understood the basic connection between movement and weight control, they often lacked specific strategies or support systems to maintain consistent activity. Without a clear plan or support, physical activity became a temporary solution rather than a consistent part of their lifestyle, reinforcing a cycle of short-lived efforts followed by relapse.

“Yes, I am a lazy person. Yes, I constantly feel guilty that I don’t exercise enough, that I don’t have a gym membership and stuff like that. But I usually walk to work quite a bit, even so” (GI2).

## Discussion

The results illustrate a complex and often fragile process of changing health behaviors among Swedish elderly men, characterized by a tension between personal ambitions and lived realities. The findings should be understood in relation to the participants’ specific life contexts, rather than as universally generalizable factors. In qualitative research, transferability is achieved through detailed descriptions of participants and settings, enabling readers to evaluate relevance to other contexts. The influence of family history and personal health conditions reflects how these men interpreted lifestyle changes in their daily lives, not causal factors applicable to all men with prediabetes.

Men expressed a desire for clear and straightforward guidance on how to change their habits; however, they also showed awareness that progress is best made through small, manageable steps. This emphasis on incremental change aligns with behavioral theories that conceptualize behavior change as a gradual, nonlinear process rather than a single decision or action, reflecting a realistic understanding of their own limitations and challenges, as well as an appreciation of the importance of sustaining healthy lifestyle changes over time. In line with previous research (28, 29), our findings highlight a close relationship between self-image and the ability to maintain healthy routines. Emotional distress, often rooted in perceived physical limitations and dissatisfaction with body image, regularly undermined the men’s motivation. The disconnect between how men viewed themselves and their current physical condition contributed to cycles of discouragement and relapse. From an SDT perspective, such negative self-perceptions may weaken perceived competence, thereby reducing intrinsic motivation and persistence over time (17, 18). These findings support previous research indicating that negative self-perceptions can hinder long-term adherence to health behaviors (30–32). Instead of showing how strong the influence is in a quantitative way, the findings reveal how men themselves understand and prioritize different factors affecting their behavior change. The importance of ambivalence, perceived limitations, and short-term thinking comes from how participants repeatedly connect these experiences to challenges in sticking with dietary changes.

These patterns can also be understood concerning gendered norms that shape how older men interpret support, responsibility, and lifestyle changes. Qualitative studies on masculinity and health have shown that norms emphasizing self-reliance, emotional restraint, and personal responsibility may lead to men’s hesitation to seek support, even when challenges are acknowledged (33, 34). Rather than explicitly rejecting help, men may frame support-seeking as unnecessary or inconsistent with their self-image, potentially delaying engagement with health-promoting services. In addition, food practices are often embedded in gendered meanings related to autonomy, pleasure, and control, which may limit the acceptability of dietary advice perceived as restrictive or externally imposed (35, 36). Together, these gendered expectations may help explain why awareness and motivation did not consistently translate into sustained behavioral change in the present study.

The men demonstrated a high level of reflective awareness regarding their personal vulnerabilities, particularly related to eating and drinking habits. However, such awareness did not consistently translate into effective coping strategies. Situational triggers such as routine coffee breaks, weekend habits, and work-related stress played a significant role in shaping behavior. This gap between awareness and action can be understood through the TTM, where individuals may remain in contemplation despite recognizing the need for change, particularly when strategies to move toward preparation or action are insufficient (16). This aligns with research suggesting that awareness alone is insufficient for sustained behavioral change when coping strategies are weak or absent (37–39). Studies on stress-related eating behaviors similarly show that individuals with strong cognitive awareness may still struggle to act in accordance with their goals under emotional strain (39). Hill et al. (37) emphasize that emotional triggers, such as workplace stress, often override rational intentions, leading to maladaptive eating patterns.

A recurring concern among men was their focus on short-term goals, especially weight loss, often driven by physical discomfort and dissatisfaction with body image. Men recognized that striving for quick results frequently led to feelings of failure when these goals were not maintained. This finding aligns with evidence that short-term interventions often yield limited, unsustainable outcomes, reinforcing negative self-perceptions and cycles of disappointment. Body image dissatisfaction has been linked to maladaptive behaviors and emotional distress, particularly when weight loss becomes the primary indicator of success (38, 39). Together, these findings underscore the importance of shifting the focus from weight-centered goals to sustainable behavioral change and psychological wellbeing. Excess body weight, especially central adiposity, is a major factor in insulin resistance and is often targeted in diabetes prevention. However, how individuals perceive and relate to their weight after a prediabetes diagnosis can affect their motivation and participation in lifestyle changes (21). From a theoretical standpoint, interventions that support autonomy, competence, and realistic goal setting may be better suited to facilitate long-term behavior change. Interventions that incorporate cognitive strategies and address body image concerns appear more effective in supporting long-term adherence (38). Previous research further indicates that patients perceive lifestyle consultations with nurses as valuable but often insufficiently tailored. Patients report a need for more time, clearer and more individualized advice, and better explanations of laboratory results and associated risks. Motivation has been shown to increase when advice is adapted to individual circumstances and preferences (40, 41). From a behavioral theory perspective, these findings resonate with the TTM (16), where men appear to fluctuate between contemplation and preparation, and with SDT (17, 18), which highlights how limited perceived competence and autonomy may undermine sustained change.

External factors such as job changes, economic constraints, and seasonal conditions were identified as barriers to maintaining healthy routines. Similar findings have been reported in research on prediabetes, where environmental and social circumstances greatly influence the success of lifestyle interventions (21). These contextual influences further illustrate how behavior change is embedded within broader social and environmental systems, which may either support or constrain individuals’ capacity to move toward action and maintenance. Previous findings highlight that work-related stress and limited resources often impede sustained behavior change (21, 42). In addition, our results indicated that life transitions, like retirement, can serve as either barriers or facilitators. Evidence suggests that retirement may offer opportunities to regain control over daily habits. (43, 44). Such transitions may represent critical windows for behavioral change, where readiness and contextual conditions temporarily align.

Although the men understood the importance of physical activity, their efforts were often described as unstructured and inconsistent. This suggests that while men were aware of the health benefits, they lacked clear strategies or structured routines to add exercise into their daily lives, indicating challenges related to both perceived competence and action planning. This may reflect common barriers like uncertainty about suitable activities or limited confidence in maintaining regular habits (32, 45). In line with previous research, men showed interest in alternative physical activities, such as walking football and senior table tennis (46, 47), indicating that socially engaging and easily accessible options may be more appealing than traditional gym settings (28, 48). Such activities may enhance motivation by supporting relatedness and enjoyment, key components of sustained engagement according to SDT (17).

### Limitations

While the study offers valuable insights into factors influencing health behavior change among men, several limitations should be acknowledged. The use of convenience sampling may have resulted in self-selection bias, as men with a particular interest in health or lifestyle change may have been more inclined to participate. This may limit the transferability of the findings. Nevertheless, the data reflected a broad range of experiences, including ambivalence, resistance, and uncertainty regarding behavior change, which strengthens the credibility of the results. A small sample size might restrict the generalizability of the findings. However, within a qualitative paradigm, the sample size is considered appropriate, as the focus lies on depth, variation of experiences, and rich contextual description, enabling readers to assess transferability. Qualitative findings are context-specific and interpretive and should therefore be viewed as illustrative rather than representative. The study relies on self-reported data, which may involve underreporting of behaviors perceived as negative, such as alcohol consumption or physical inactivity. Nonetheless, some men openly discussed such behaviors, and these accounts were included in results. The interviewer effects or the interview environment could have impacted responses. The men might have shaped their answers based on what they thought the interviewer expected or felt comfortable sharing, especially about sensitive topics like body image, self-worth, or perceived failure. The digital interviews might limit the observation of nonverbal cues and may be interrupted by technical problems such as unstable internet, delayed audio, or poor video quality, all of which can affect the flow of conversation and expression. Men might also feel less at ease discussing sensitive topics online, which, in turn, could reduce the depth of the data. Finally, the absence of longitudinal follow-ups limits the ability to evaluate how men’s attitudes and behaviors may change over time. Since health behavior change is often dynamic, a cross-sectional snapshot may overlook important fluctuations or turning points. The novelty of this study is in combining established qualitative findings on prediabetes-related ambivalence with a gender-sensitive analysis of how older men manage lifestyle advice, seek support, and deal with behavioral setbacks within masculinity norms.

## Conclusion

Health behavior change among men with prediabetes is not a straightforward ride. It is a fragile and dynamic journey influenced by personal doubts, emotional triggers, and external circumstances. The findings suggest that men with prediabetes commonly experience a tension between understanding health advice and translating it into sustained action. Participants described ambivalence, emotional responses, and contextual constraints as central challenges in their efforts to change dietary habits. At the same time, the findings reveal signs of progression. Some men actively sought support, reflected on their experiences, and learned from both successes and setbacks, gradually developing a more realistic understanding of their capacity for change. The men also expressed interest in socially engaging and accessible forms of physical activity, suggesting that interventions should extend beyond traditional approaches. Life transitions, such as retirement, may provide opportunities for positive change, whereas job stress and environmental or financial constraints represent significant challenges that should be addressed within dietary and lifestyle counseling. These conclusions are based on participants’ stories and the analytic patterns found across interviews, rather than on quantitative data.

### Implications

Our findings suggest that interventions aimed at promoting health behavior change should adopt a comprehensive and person-centered approach that addresses both emotional and environmental factors. Providing emotional support and fostering self-compassion seem essential, especially in reducing the adverse effects of self-labelling and feelings of failure that often follow setbacks. Encouraging individuals to view relapse as a regular part of the change process can help build resilience and maintain motivation. Furthermore, interventions should help the men set realistic, long-term goals that extend beyond weight loss and focus on overall wellbeing. Recognizing and managing personal triggers, such as routine snacking or work-related stress, can help develop healthier routines. Significantly, providing accessible and enjoyable physical activities tailored to individual preferences and abilities, like walking football or senior table tennis, may boost motivation and commitment. By understanding the emotional complexities and situational barriers involved in health behavior change, health promotion efforts can more effectively support individuals in creating sustainable habits and improving their quality of life.

## Data Availability

The raw data supporting the conclusions of this article will be made available by the authors, without undue reservation.
